# Hair Cortisol Concentrations in Children: A Longitudinal Analysis Across Childhood

**DOI:** 10.1002/dev.70154

**Published:** 2026-04-19

**Authors:** Miguel Ángel Baos‐González, Javier De Echarri‐Lorente, María Ángeles García‐León, Raquel González‐Pérez, María Isabel Peralta‐Ramírez

**Affiliations:** ^1^ Centro de Investigación Mente, Cerebro Y Comportamiento (CIMCYC) Universidad de Granada Granada Spain; ^2^ Facultad de Psicología, Departamento de Personalidad, Evaluación y Tratamiento Psicológico Universidad de Granada Granada Spain; ^3^ Facultad de Psicología Universidad de Sevilla Seville Spain; ^4^ Facultad de Farmacia, Departamento de Farmacología Universidad de Granada Granada Spain; ^5^ Centro de Investigación Biomédica en Red de Enfermedades Hepáticas y Digestivas (CIBERehd) Madrid Spain; ^6^ Instituto de Investigación Biosanitaria Granada Spain

**Keywords:** children, hair cortisol concentration, HPA axis, longitudinal study, percentiles

## Abstract

Hair cortisol concentration (HCC) reflects long‐term cortisol exposure and is commonly used as a biomarker of hypothalamic–pituitary–adrenal axis activity. Although increasingly applied in pediatric populations, developmental changes in HCC and potential sex differences remain unclear. This longitudinal study examined HCC across childhood and the influence of sociodemographic and perinatal factors. A total of 212 mother–child dyads participated. Sociodemographic and perinatal data were collected during pregnancy and postpartum. Offspring HCC (pg/mg) was assessed at birth, 6 months, and annually from 1 to 7 years. HCC decreased with age, with the steepest decline occurring in early childhood and greater stability observed between 4 and 7 years of age. Sex differences emerged only at 3 years, with higher HCC in females. Standardized birth height was negatively associated with newborn HCC; maternal BMI and weight before and during pregnancy were negatively associated with HCC at 1–2 years; and gestational week at delivery was positively associated with HCC at 3 years. Descriptive HCC percentiles were calculated for newborns, at 6 months, and at ages 1–3 years. These findings illustrate the developmental trajectory of HCC across childhood and provide insights into sociodemographic and perinatal factors associated with children's HCC.

## 1 Introduction

Cortisol is a glucocorticoid hormone and the main hormonal product of the hypothalamic–pituitary–adrenal (HPA) axis. This hormone is essential to the functioning of the metabolic, immune, and neurological systems (Gao et al. 2022; Sapolsky 2004; Spencer and Deak 2017). The HPA axis plays a major role in the stress response: indeed, the hypothalamus perceives stressful stimuli and releases corticotropin‐releasing hormone (CRH), triggering the adrenocorticotropic hormone (ACTH) secretion from the anterior pituitary, ultimately leading to glucocorticoid production by the adrenal glands (Spencer and Deak 2017).

Thus, a properly functioning HPA axis is essential for restoring homeostasis and maintaining mental and physical health. Prolonged exposure to stress can alter the adaptive response of the HPA axis to environmental threats, which has been associated with higher risk of depression and other stress‐related mental disorders (McEwen 2017; Wang et al. 2024). In their systematic review, Malisiova et al. (2021) found HPA axis activity differences based on hair cortisol concentration (HCC) levels in patients with depression, posttraumatic symptoms disorder, general anxiety disorder, and schizophrenia compared to controls.

Cortisol is the most widely used glucocorticoid to evaluate stress response and is widely used as an indicator of HPA axis–related activity. It has been measured in humans using urine, plasma, saliva, and hair samples. However, HCC has been clearly recognized as the best method for assessing long‐term cortisol exposure, as it can reflect cortisol accumulated over weeks or months, whereas blood, saliva, or urine can signal cortisol produced only in the previous moments, hours, or days. These methods present other disadvantages, such as the influence of confounders—for example, acute stressors or circadian rhythms—their invasive nature, as well as collection and storage difficulties. The HCC method is thus gaining popularity because it is noninvasive, painless, easy to collect and store, and able to provide an index of long‐term cortisol exposure (Li et al. 2023; Malisiova et al. 2021; Stalder et al. 2017). More specifically, it has been recognized that HCC allows quantifying the accumulation of unbound free circulating cortisol produced and incorporated into growing hair over time, providing a long‐term indicator of systemic cortisol levels (Russell et al. 2012; Stalder and Kirschbaum 2012). Therefore, HCC is considered an optimal retrospective biomarker of long‐term cortisol exposure (Garcia‐León et al. 2018; Li et al. 2023; Russell et al. 2012). It is also ideal to use in large cohort studies, as it is understood to reflect cortisol variations due to long‐term psychological stress (Bryson et al. 2021; Malisiova et al. 2021). Indeed, hair grows at a fairly consistent rate of approximately 1 cm per month, so the 1‐cm segment closest to the scalp approximates 1 month of cortisol concentration. The second closest centimeter approximates cortisol production in the previous month, and so on (Wennig 2000). Hence, by collecting a 3‐cm hair segment, researchers can obtain accumulated hair cortisol from approximately the three previous months as an indicator of long‐term HPA axis–related activity.

HCC has proven to be a useful measure in the adult population as a biomarker of long‐term cortisol exposure and, therefore, as a valuable instrument for different purposes, that is, assessing the contribution of prolonged stress exposure, evaluating therapeutic outcomes, predicting mental disorder relapses, or selecting optimal patient therapy (Li et al. 2023; Malisiova et al. 2021). Furthermore, Li et al. (2023) highlighted its applicability as a biomarker of long‐term cortisol exposure in pediatric populations, while several other studies have found promising results in children. For example, HCC has been inversely associated with externalizing problems, which, in turn, have been associated with emotional abuse (C. Chen and Duan 2023). Moreover, regarding family and sociodemographic data, HCC serves as a biological indicator of socioeconomic disparities: families experiencing financial difficulties exhibit higher cortisol levels (Ling et al.^2019^). Child HCC has also been shown to be positively related to mother HCC (Dauegaard et al. 2020; Romero‐González et al. 2018). In a different vein, HCC levels have equally been linked to various forms of psychopathology. Brænden et al. (2023) found elevated HCC levels in children diagnosed with disruptive mood dysregulation disorder (DMDD) and other psychological disorders compared to healthy peers. Similarly, Gao et al. (2022) found higher HCC levels in autism spectrum disorder (ASD) patients. Studies are still lacking, however, on HCC measures in relation to other disorders known to present a different HPA functioning. This is the case of attention‐deficit/hyperactivity disorder (ADHD), which has been associated with lower basal salivary cortisol levels, especially in the morning (Chang et al. 2021). To summarize, the use of HCC as a biomarker of cortisol exposure enables researchers to explore links between social, biological, and psychological determinants.

Although HCC is gaining popularity in the pediatric population, reference values comparable to those available in adults have not yet been established (Garcia‐León et al. 2018), and the influence of several factors on HCC during childhood remains insufficiently clarified. Previous research highlights sex and pubertal stage as HCC‐mediating variables (Gray et al. 2018; Wang et al. 2024; White et al. 2017). Moreover, in their systematic review, Gray et al. (2018) highlight the possible influence of anthropometry (body mass index and waist circumference) and the absence of influence of hair wash frequency and use of hair treatments (if proximal hair segments are used), suggest socioeconomic status as a confounder with preliminary evidence, and note the conflicting evidence of age. Besides, further research is required to clarify the role of perinatal variables in short‐ and long‐term HCC.

Specifically, regarding age, there is conflicting evidence concerning the presence and direction of its relationship with HCC in children. In the systematic review by Gray et al. (2018), 14 studies were identified on this relationship, of which 10 found no relation with age and four did determine significant differences. Of the latter, two encountered positive relations (Noppe et al. 2014; White et al. 2017), while two studies obtained negative relations (Dettenborn et al. 2012; Karlén et al. 2013). Following the review by Gray et al. (2018), we detected four more studies analyzing this relationship. Kao et al. (2018) found no relation, Simmons et al. (2019) as well as Ling et al. (2019) identified a negative correlation, and Rickmeyer et al. (2017) found a positive correlation. All these studies, however, present important limitations because their main objective was not to examine the HCC–age relationship. Most used age ranges that are either too narrow or too extensive, or the samples were small in size. Moreover, the age ranges differ among the studies, making it difficult to extract conclusions. Research in this field requires clarification and analysis within specific age ranges. Longitudinal studies including children of various ages and precise measurements are particularly important to conduct (Gray et al. 2018). To the best of our knowledge, only one study meets this requirement and uses a large sample: Karlén et al. (2013) found that HCC decreased between 1 and 8 years of age (1 > 3 > 5 > 8), and that that decrease was steepest early on. Taken together, the current evidence remains inconclusive, highlighting the need for longitudinal studies with sufficient sample size and well‐defined age ranges to clarify how HCC changes across childhood.

Therefore, the main aim of the present longitudinal research was to examine HCC across childhood in a large sample of healthy Spanish children, while also exploring associations with sociodemographic and perinatal variables. In addition, descriptive age‐specific HCC percentiles were calculated to illustrate the distribution of cortisol levels observed in early childhood within this cohort. The choice of a longitudinal study was based on the importance of tracking changes in HCC levels over time and understanding how this stress biomarker varies during childhood. Importantly, this manuscript focused only on prepubertal children.

## 2 Material and Methods

### 2.1 Participants

**FIGURE 1:**
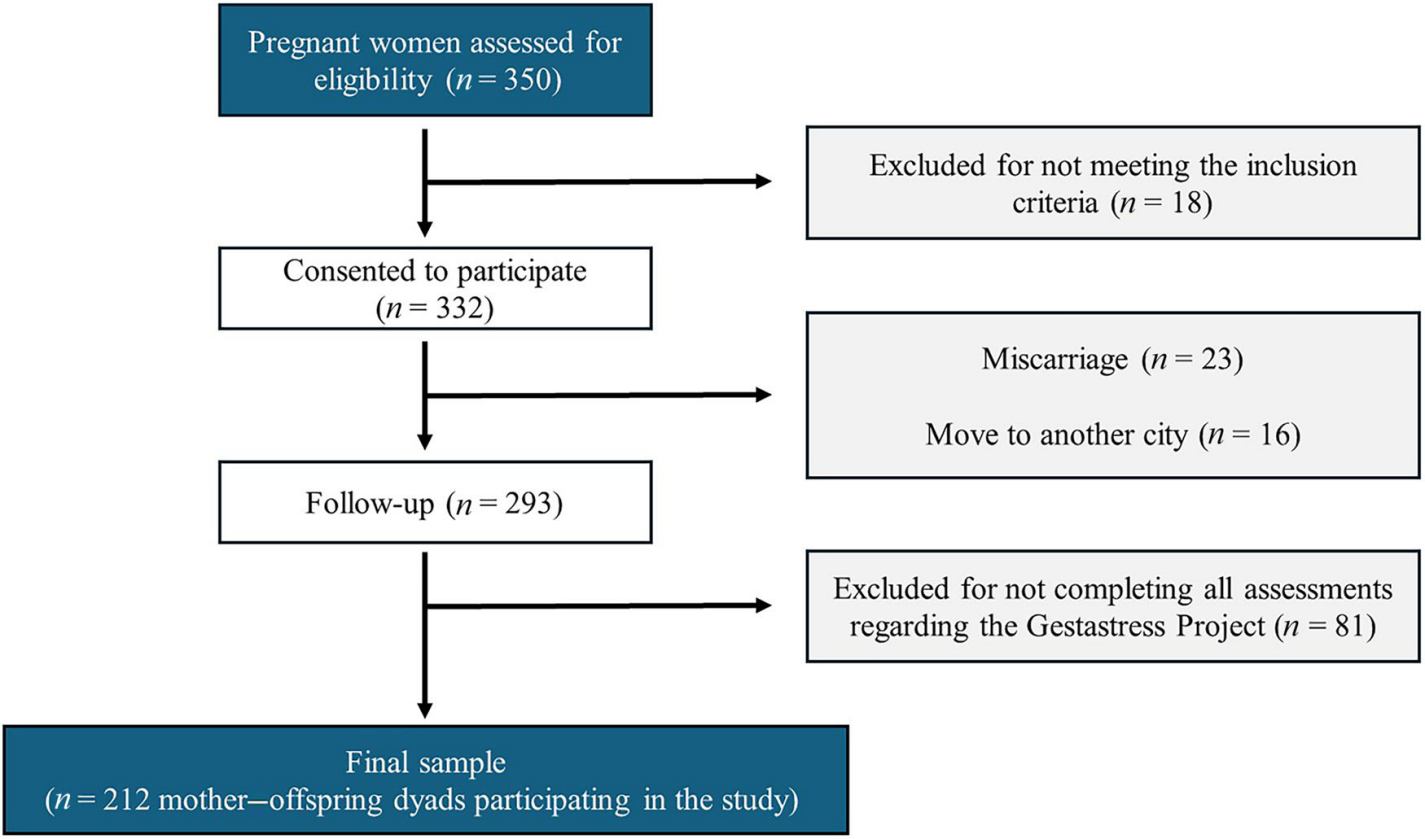
Flowchart of pregnant women's recruitment process. Schematic representation of the recruitment process for the present study, starting from initial eligibility assessment (*n* = 350 pregnant women) through to the final analytical sample (*n* = 212 mother–offspring dyads). The diagram specifies reasons for exclusion, including failure to meet inclusion criteria, miscarriage, relocation, and incomplete assessments during the follow‐up period.

The participants were recruited over several years, as part of the “Gestastress–Childstress” cohort. A sample of 350 healthy Spanish pregnant women, mainly from the city of Granada (Spain), were informed of this study during their prenatal hospital visits. Infants thus became study participants from the moment they were born. A total of 18 women were excluded because they failed to meet the inclusion criteria, and a total of 332 signed the informed consent form. Twenty‐three had a miscarriage, and 16 left the study because they moved to another city. A further 81 were excluded because they did not complete all assessments regarding the Gestastress Project. A final total of 424 participants, that is, 212 mothers and their children, started participating in this longitudinal study (Figure 1).

**FIGURE 2:**
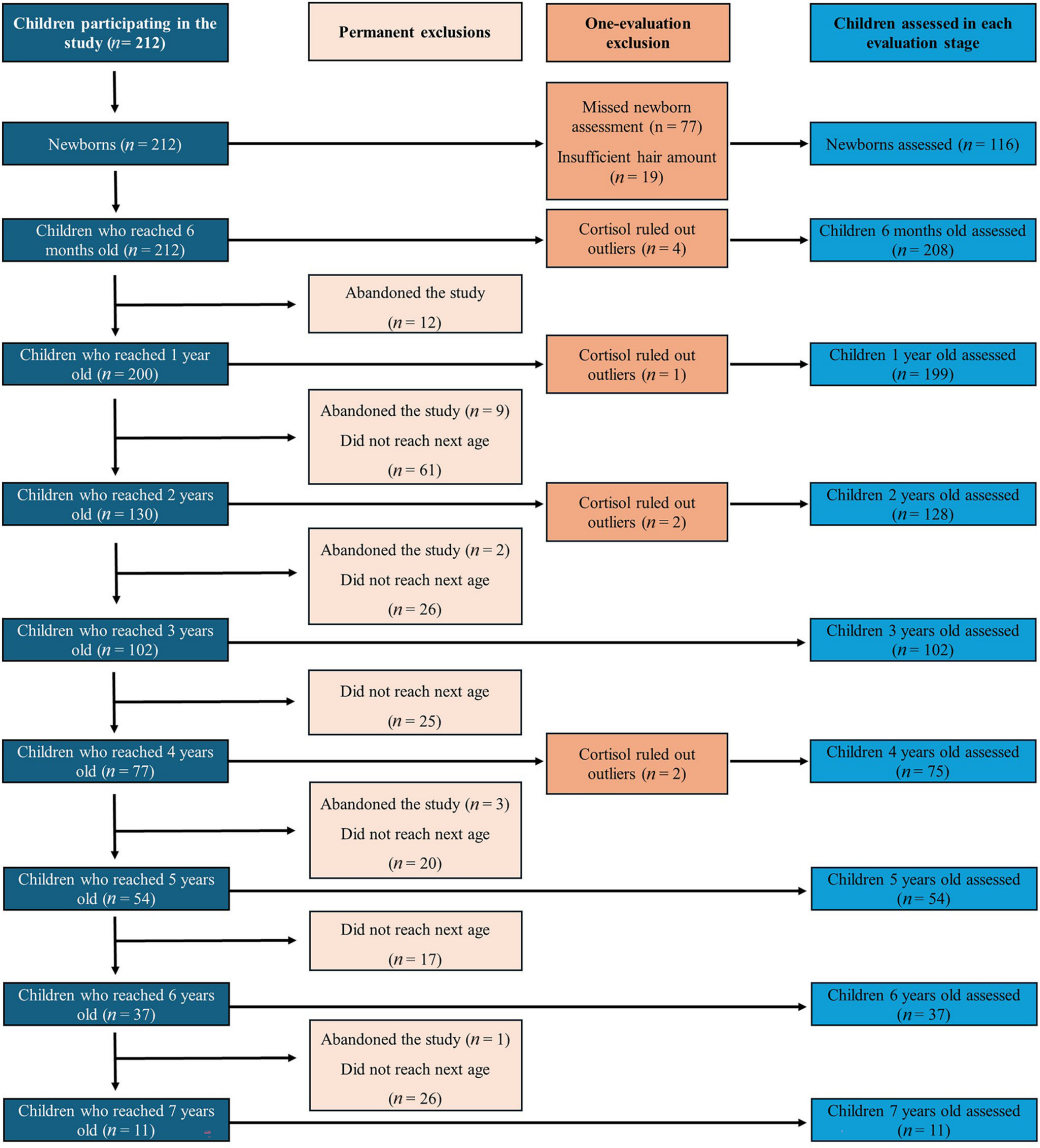
Flowchart of children sample throughout the longitudinal follow‐up. Schematic overview of the participants’ follow‐up from birth to age 7. The diagram illustrates the progression of participants through each developmental stage, detailing permanent exclusions (study withdrawal and age‐out criteria) and one‐evaluation exclusions (missed assessments, insufficient hair amount in the sample, or cortisol outliers). The final column indicates the number of children who were successfully assessed at each time point, ranging from newborns to 7 years of age.

The child sample consisted of 212 newborns who were followed up in the longitudinal study. Nevertheless, sample size diminished as participant age increased, leading to sample size differences by age and age group. The main reasons for the participants’ attrition during the follow‐up were experimental death, children who entered the study later or who missed an appointment (i.e., during the newborn period), and HCC outliers (see Section 2.4) as shown in Figure 2.

Regarding the selection of pregnant women, the exclusion criteria were as follows: being aged under 18 years; having a physical or physiological illness; using glucocorticoids or medication known to alter glucocorticoids metabolism; and psychiatric disease. The exclusion criteria for children consisted of having any form of disability and using glucocorticoids.

The Human Studies Ethics Committee of the University of Granada (Spain) approved the study (968/CEIH/2019), which was conducted in conformity with the American Psychological Association's (APA) Ethical Principles of Psychologists and Code of Conduct. The sample was collected in accordance with the 1975 Helsinki Declaration and its subsequent revisions. After reading the information file, each participant signed the consent form for themselves and their offspring to participate in the study.

### 2.2 Instruments

#### 2.2.1 Structured Interview

The interviews were conducted at 10 different times (see Section 2.3). In the prenatal phase, the occurrence of any highly stressful event in the previous 3 months (yes/no), maternal height, employment status (employed/unemployed), alcohol intake (yes/no), smoking (yes/no), household income (<1000€; 1000–2000€; 2000–3000€; 3000–4000€; >4000€), pregestational weight, and weight in the first trimester of pregnancy were recorded. The assessment of stressful events encompassed events from multiple domains (e.g., work, family, housing). Later, at the first postnatal visit, the “newborn evaluation” was conducted, during which the gestational week of delivery and the type of delivery (coded as “spontaneous delivery,” “induced delivery,” or “caesarean section”) were recorded, along with the baby's sex, weight, height, cephalic perimeter, and “firstborn” or “not‐firstborn” status. Educational attainment was divided into “secondary school or less” and “university.” In subsequent assessments, that is, when the children were aged 6 months or more, only the current age was asked.

#### 2.2.2 Analysis of HCCs of the Children

**FIGURE 3:**
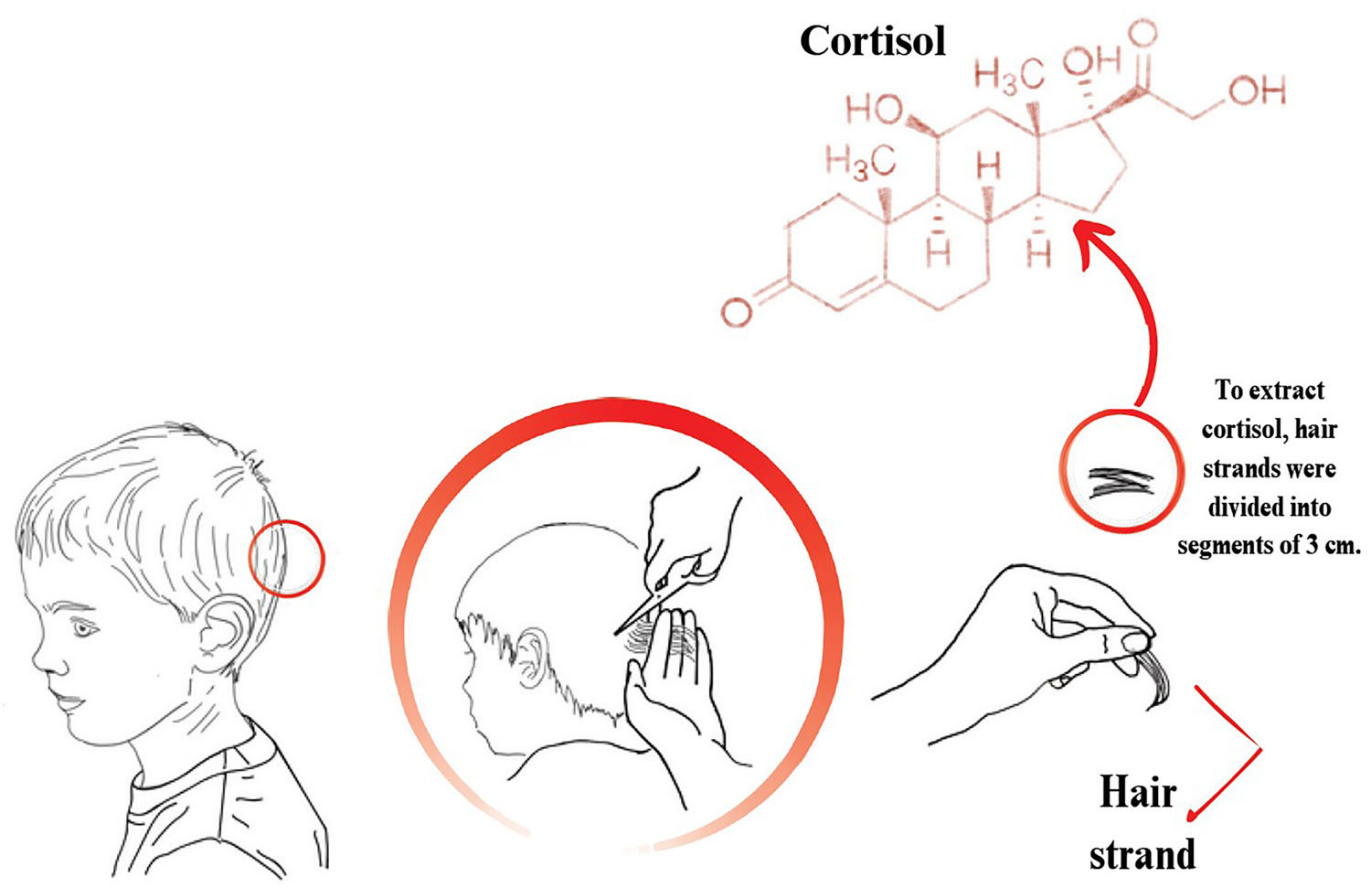
Artwork illustrating the hair sample collection process. Hair strands were collected from the posterior vertex of the scalp, as indicated by the red circle. Samples were obtained by cutting the hair as close to the scalp as possible. For the extraction of hair cortisol concentration (HCC), strands were subsequently divided into 3‐cm segments to reflect retrospective hormonal exposure. The chemical structure of the cortisol molecule is shown in the upper right.

The hair samples consisted of locks of approximately 150 strands of hair taken from the posterior vertex, cut as closely to the scalp as possible (Figure 3). Each sample was then wrapped in aluminum foil to protect it from light and humidity, and stored in an envelope at room temperature. Later, the samples were analyzed in the Department of Pharmacology at the University of Granada, Spain. In our study, 3‐cm long hair samples were collected to measure HCC from a 3‐month period (assuming an average growth rate of 1 cm per month). The samples were first washed twice in isopropanol to remove any cortisol from the outside of the hair shaft that had been deposited from sweat or sebum. Then, they were dried for 3 days at room temperature and in the dark. Third, samples were weighed and ground to a fine powder using a ball mill (Bullet Blender Storm, Swedesboro NJ, USA) to break up the hair's protein matrix and to increase the surface area for extraction. The weight of processed samples ranged between 10 and 40 mg. Later, samples were incubated in 1 mL of HPLC‐grade methanol for 72 h at room temperature in the dark with constant inversion using a rotator, a duration that allows extraction of most of the cortisol from hair samples (Z. Chen et al. 2013). After incubation, the samples were centrifuged and the supernatant was evaporated until completely dry using a vacuum evaporator for 2 h at 30°C (Centrivac, Heraeus, Hanau, Germany). This extract was then reconstituted in 150 µL of phosphate‐buffered saline (PBS) at pH 8.0. The reconstituted sample was immediately frozen at −20°C for later analysis (Z. Chen et al. 2013; Kirschbaum et al. 2009; Meyer et al. 2014). Finally, the HCC (pg/mg) of each sample was measured using the Cortisol Salivary ELISA kit (Alpco Diagnostics) with PBS at pH 8.0. The manufacturer directions for correct usage were provided with the reagent. The cross‐reactivity, as reported by the manufacturer, is as follows: prednisolone 13.6%, corticosterone 7.6%, deoxycorticosterone 7.2%, progesterone 7.2%, cortisone 6.2%, deoxycortisol 5.6%, prednisone 5.6%, and dexamethasone 1.6%. No cross‐reaction was detected with DHEAS and tetrahydrocortisone.

#### 2.2.3 Composite Socioeconomic and Psychosocial Adversity Score

This score was created to capture cumulative exposure to adversity, as recommended in prior meta‐analytic research on infants’ HCC (Bryson et al. 2021). The score was derived from dichotomous socioeconomic, psychosocial, and perinatal indicators obtained from the pregnancy and postpartum interviews, yielding a total score ranging from 0 to 12, with higher values indicating greater adversity. The selection of items was based on previously published indices (Bhopal et al. 2019; Bryson et al. 2019, 2021) and adapted to the variables available in the study. The included indicators were as follows: mother's highest level of education is elementary school, mother is unemployed, single/separated/divorced mother not living with partner, young pregnancy (<23 years), alcohol consumption during pregnancy, smoking during pregnancy, very low household income (<1000€), presence of highly stressful events during pregnancy, child born early (<37 weeks), mother's prepregnancy obesity (BMI > 30), caesarean section delivery, and low birth weight (<2500 g).

### 2.3 Procedure

In accordance with the longitudinal design, the children were followed up from maternal pregnancy to 7 years of age. The study began in the pregnancy period, when the women were informed about the objective and the procedure to follow during pregnancy and then later, before signing the informed consent form. The women then completed the first version of the semistructured interview questionnaire. The next version of the interview was completed after delivery (newborn evaluation stage), and the subsequent ones were completed when the children reached the defined evaluation stages (aged 6 months and then 1, 2, 3, 4, 5, 6, and 7 years). Hair samples were collected from the posterior vertex of the child's head at each stage. On average, the first meeting with the pregnant woman lasted approximately 15 min, the newborn evaluation during the postnatal visit around 20 min, and the rest of the evaluations 5 min. See Section 2.2.1 for details on the variables assessed at each stage.

### 2.4 Statistical Analyses

First, the Kolmogorov–Smirnov test was implemented to analyze normal data distribution. Since the results indicated nonnormality, all values were log‐transformed for statistical analyses. Untransformed HCC values in picograms per milligram were, however, reported for descriptive purposes. HHC outliers of more than 3 standard deviations (SDs) were excluded (Field 2024).

Then, birth weight and height were converted into standardized deviation scores (SDSs) based on the Intergrowth reference standards (Villar et al. 2014) to obtain normalized measures accounting for gestational age and sex. Descriptive statistics consisted of means (*M*) and SD for normally distributed variables and relative frequencies for categorical variables. HCC differences according to age were analyzed using ANOVA. When significant HCC differences were found between children of different ages, they were separated into different age groups, and when HCC did not vary across more than two consecutive ages, these age groups were combined. In addition to ANOVA tests, we fitted linear mixed‐effects models—including linear and quadratic age terms—to characterize the developmental trajectory of HCC across childhood. We also estimated a separate model restricted to ages 4–7 to evaluate whether this period represented a statistically stable phase of HCC.

Potential nonrandomness of missing data was examined by comparing children who provided at least one HCC measurement between 4 and 7 years of age with those who did not reach this follow‐up period. Groups were compared on sociodemographic, maternal, and perinatal variables using independent‐samples *t* tests for continuous variables and chi‐square tests for categorical variables.

Additionally, the Pearson correlation coefficients (PCCs) and simple linear regression were calculated in order to identify all potentially significant HCC determinants. The Student's *t* test and univariate ANOVAs with a Bonferroni post hoc test were also performed in order to test group differences according to sociodemographic and perinatal variables.

Finally, HCC percentiles were calculated for newborns, at 6 months, and at 1–3 years using the weighted average method and the HCC untransformed values. The 4‐ to 7‐year group was excluded from the percentile analyses due to insufficient sample sizes across this age range. Statistical analyses were performed using SPSS 23.0 (IBM Corp., Armonk, NY, USA).

## 3 Results

### 3.1 Sample Characteristics

The final child sample spanned the different ages as follows: 116 newborns, 208 infants aged 6 months, 199 aged 1 year, 128 aged 2 years, 102 aged 3 years, 75 aged 4 years, 54 children aged 5 years, 37 aged 6 years, and 11 aged 7 years. HCC outliers were excluded from the analysis (6 months: *n* = 4; 1 year: *n* = 1; 2 years: *n* = 2; 4 years: *n* = 2). A final total of 930 hair samples were collected from birth until the age of 7 years. Relevant sociodemographic child data are shown in Table 1.

**TABLE 1:** Sociodemographic and perinatal variables.

Variable	*n*	% / *M* (SD)
**Mothers**	208	
Age (years)		32.6 (4.5)
Educational attainment		
Secondary school or less		25.9%
University		74.1%
Civil status		
Married		59.8%
Unmarried partner		40.2%
Employment status		
Employed		78.6%
Unemployed		21.4%
Household income		
<2000€		21.8%
>2000€		78.2%
Occurrence of highly stressful events in the previous 3 months		
Yes		34%
No		66%
Smoking during pregnancy		
Yes		6.1%
No		93.9%
Alcohol intake during pregnancy		
Yes		1.1%
No		98.9%
Pregestational BMI		23.4 (4.5)
BMI in first trimester of pregnancy		26.1 (4.8)
Contacted in first trimester of pregnancy		
Yes		74.8%
No		25.2%
First pregnancy		
Yes		45.5%
No		54.5%
Type of delivery		
Spontaneous delivery		56.6%
Induced delivery		41.2%
Caesarean section		2.2%
**Newborns**	116	
Sex (male / female)		52.6% / 47.4%
Age (in days)		16.0 (8.7)
Birth weight (g)		3262 (411)
Birth weight SDS		0.05 (0.93)
Birth height (cm)		50.6 (2.94)
Birth height SDS		1.01 (1.30)
Cephalic perimeter		34.4 (2.1)
Week of birth		39.5 (1.2)
**6 months**	208	
Sex (male / female)		46.6% / 53.4%
Age (in months)		6.5 (0.7)
**1 year**	199	
Sex (male / female)		47.2% / 52.8%
Age (in months)		12.5 (0.8)
**2 years**	128	
Sex (male / female)		44.1% / 55.9%
Age (in months)		24.5 (1)
**3 years**	102	
Sex (male / female)		42.2% / 57.8%
Age (in months)		36.5 (0.7)
**4 years**	75	
Sex (male / female)		44% / 56%
Age (in months)		48.8 (0.9)
**5 years**	54	
Sex (male / female)		48.1% / 51.9%
Age (in months)		60.7 (0.9)
**6 years**	37	
Sex (male / female)		43.2% / 56.8%
Age (in months)		72.5 (0.5)
**7 years**	11	
Sex (male / female)		36.4% / 63.6%
Age (in months)		85 (1)

Abbreviation: SDS, standardized deviation score.

In the case of the mothers, most pregnant women were recruited in the first trimester of pregnancy (74.8%), had higher education (74.1%), had spontaneous delivery (56.6%), and were married (59.8%). Relevant mother sociodemographic characteristics are included in Table 1. Moreover, the composite socioeconomic and psychosocial adversity score had a mean of 0.64 (SD = 0.85).

Analyses further examining participant attrition revealed no significant differences between retained and non‐retained participants in maternal age, educational attainment, household income, civil status, pregestational BMI, BMI in the first trimester of pregnancy, recruitment during the first trimester of pregnancy, primiparity, occurrence of highly stressful events during pregnancy, or child‐related variables, including sex, head circumference, birth length, birth weight, and week of delivery. However, a significant difference was found for type of delivery, *χ* ^2^(2) = 10.19, *p* = 0.006, such that children who provided at least one HCC measurement between 4 and 7 years of age were more likely to have been born via caesarean section or induced delivery compared with those who did not reach this age range.

### 3.2 HCC Group Differences According to Age

The ANOVA analyses showed statistically significant HCC differences between children of most ages, from newborns to 3 years, but no differences between children aged 4–7 years (see Table S1). Each year thus constituted an age group until 4 years of age, which was when cortisol levels stabilized. Subsequently, children aged 4–7 years were combined forming a single group.

To further examine this pattern, linear mixed‐effects models were fitted. These models showed a significant linear effect of age [*F*(1, 771.74) = 10.11, *p* < 0.001] and a significant quadratic effect [*F*(1, 838.02) = 54.77, *p* < 0.001], confirming a steep early decline in HCC followed by a progressive flattening of the trajectory. When restricting the model to children aged 4–7 years, age was not a significant predictor [*F*(1, 41.53) = 0.439, *p* = 0.511], indicating no systematic age‐related variation within this period.

However, the smaller sample size in 4–7 years may have limited statistical power, contributing to the nonsignificant differences.

**FIGURE 4:**
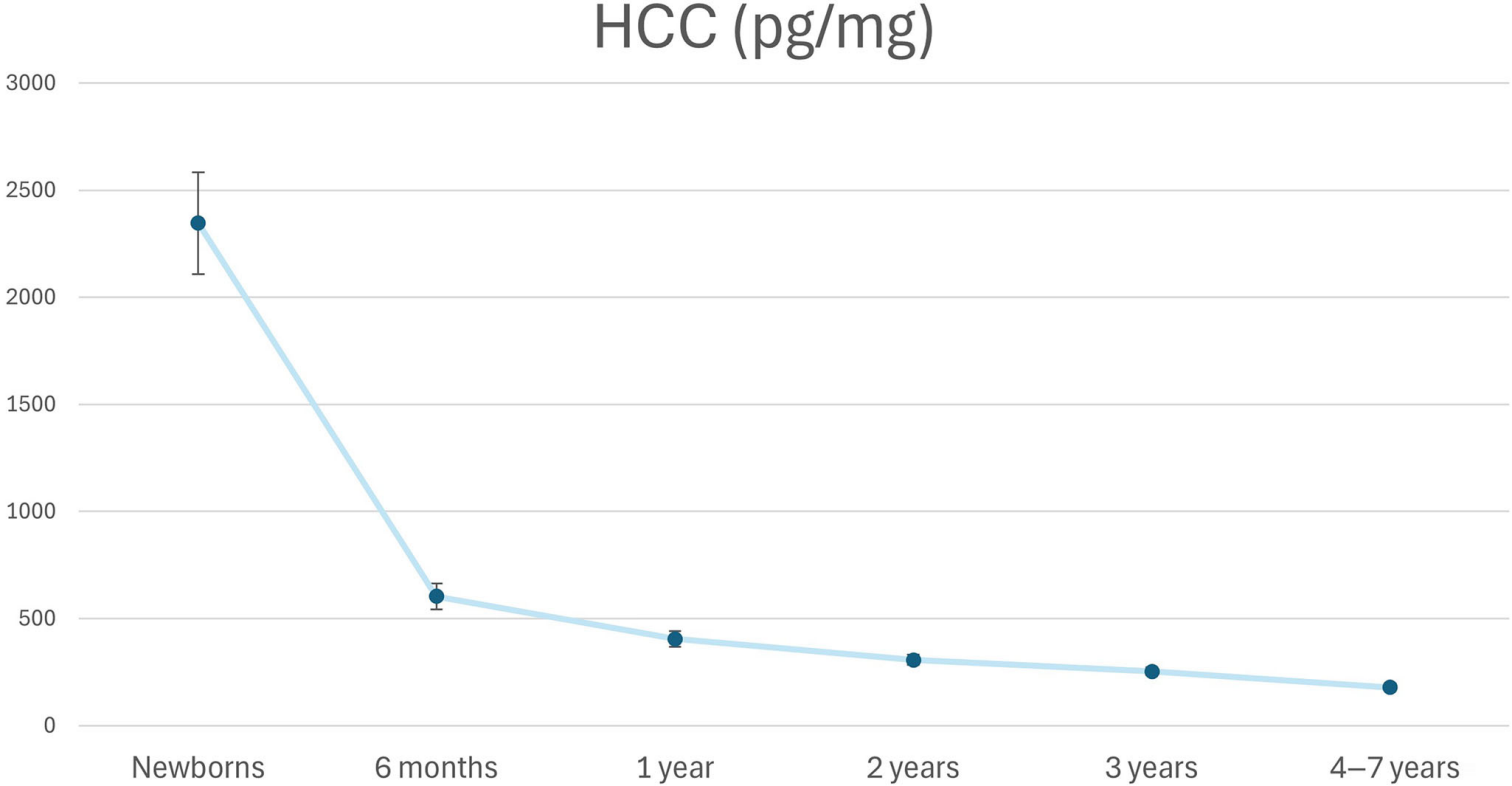
Mean HCC levels for each age group. Points represent mean untransformed HCC levels (pg/mg) for each developmental stage (newborns, 6 months, and 1, 2, 3, and 4–7 years), with error bars indicating the 95% confidence intervals (CIs). The trajectory illustrates a progressive decline in HCC throughout childhood, with the most pronounced reduction occurring during the first 6 months of life.

The age group division was based on our results as previously explained and is supported by the literature (Gray et al. 2018). Finally, six different groups were found according to HCC variability between the different age groups. For illustrative purposes, the untransformed mean HCC (pg/mg) for each group is reported in Figure 4. In addition, age, sex distribution, and untransformed HCC values are included per age group in Table 2.

**TABLE 2:** Sociodemographic and HCC information per age group.

Age group	*n*	Age in months^a^	Untransformed HCC values	Sex		Untransformed HCC values by sex
		Mean (SD)	Mean (SD)		%	Mean (SD)
Newborns	116	16 (8.7)^a^	2346.4 (2562.4)	Male	52.6	2076.3 (2451.4)
				Female	47.4	2481.4 (2426.7)
6 months	208	6.5 (0.7)	603.2 (871.2)	Male	46.6	697.9 (1073.8)
				Female	53.4	556.5 (722.1)
1 year	199	12.6 (0.9)	405.2 (512.7)	Male	47.2	410.6 (705.1)
				Female	52.8	399.2 (277.5)
2 years	128	24.6 (1.1)	306.9 (267.9)	Male	44.1	322.8 (323.8)
				Female	55.9	296.1 (219.9)
3 years	102	36.6 (0.8)	253.6 (182.2)	Male	42.2	206.6 (17)
				Female	57.8	287.8 (181.8)
4–7 years	177	61.3 (11)	178.5 (150.7)	Male	44.7	200.5 (176.5)
				Female	55.3	178.1 (130.1)

*Note:* Untransformed HCC values are shown in pg/mg.

^a^ Days postpartum.

### 3.3 Confounding and Mediating HCC Variables

We examined the relationship between cortisol and the confounding variables: sex, educational attainment, household income, occurrence of highly stressful events during pregnancy, composite socioeconomic and psychosocial adversity score, birth weight, birth height, cephalic perimeter, week of birth, type of delivery, and body mass index (BMI) before pregnancy and BMI during pregnancy. No relationship was found in the 6 months and 4–7 years age groups between HCC and any of the variables analyzed. The variables educational attainment, household income, composite socioeconomic and psychosocial adversity score, cephalic perimeter, and type of delivery showed no relationship with HCC in any of the age groups.

However, among newborns, lower HCC levels were observed in children whose mothers reported highly stressful events during pregnancy (Welch's *F* = 5.13, *p* = 0.027), with a moderate effect size (Cohen's *d* = −0.49). Birth height SDS was also negatively associated with HCC (*r* = −0.49; *p* = 0.013). At later ages, no statistically significant associations were observed between prenatal stressful events and HCC, with very small effect sizes between 6 months and 3 years (Cohen's *d* = −0.05 to 0.12), whereas in children aged 4–7 years, a small effect was observed that approached statistical significance (Cohen's *d* = −0.33; *p* = 0.051).

Moreover, in infants aged 1 year, mother BMI before pregnancy (*r* = −0.20; *p* = 0.012), weight before pregnancy (*r* = −0.20; *p* = 0.013), BMI in the first trimester of pregnancy (*r* = −0.20; *p* = 0.014), and weight in the first trimester of pregnancy (*r* = −0.18; *p* = 0.025) were negatively related to HCC levels. The same relations were found regarding mother's weight in the 2 years of age group and HCC. Mother's BMI before pregnancy (*r* = −0.21; *p* = 0.040), weight before pregnancy (*r* = −0.22; *p* = 0.027), BMI in the first trimester of pregnancy (*r* = −0.24; *p* = 0.021), and weight in the first trimester of pregnancy (*r* = −0.25; *p* = 0.015) correlated negatively with offspring HCC.

At 3 years of age, HCC levels were influenced by birth of week (*r* = 0.32; *p* = 0.017). Significant differences were also found at 3 years of age according to sex using Welch's *F* (*F* = −2.49; *p* = 0.017), with females exhibiting significantly higher HCC levels than males. Differences by sex were only found in that age group.

### 3.4 HCC Percentiles for Children

Descriptive percentiles were calculated for the next age groups: newborns and 6‐month‐, 1‐year‐, 2‐year‐, and 3‐year‐olds (Table 3).

**TABLE 3:** HCC percentiles for child age groups.

	*n* = 116	*n* = 208	*n* = 199	*n* = 128	*n* = 102
HCC percentiles	Newborns	6 months	1 year	2 years	3 years
Percentile 5	437.95	60.08	33.25	46.11	34.92
Percentile 10	539.65	100.79	78.14	76.81	51.60
Percentile 15	561.65	158.65	107.29	104.78	60.41
Percentile 20	578	196.01	136.11	118.08	84
Percentile 25	723.75	226.03	171.61	122.21	111.95
Percentile 30	1006.65	252.36	190.82	146.77	131.93
Percentile 35	1170.35	311.33	233.14	165.77	147.27
Percentile 40	1458.63	389.50	267.79	177.60	177.30
Percentile 45	1559.55	416.48	295.79	201.84	198.71
Percentile 50	1668.90	463.25	324.09	213.81	203.68
Percentile 55	1764.27	515.33	380.95	234.24	219.81
Percentile 60	1879.32	569.15	457.84	269.83	253.56
Percentile 65	2341.41	588.58	487.62	312.59	295.29
Percentile 70	2710.13	627.43	522.36	368.70	335.58
Percentile 75	3059.42	637.29	544.16	433.14	362.33
Percentile 80	3623.47	662.80	587.49	526.24	418.64
Percentile 85	3960.47	691.82	619.79	564.52	463.64
Percentile 90	4575.66	840.83	671.27	622.95	556.75
Percentile 95	4989	1543.85	841.74	706.72	617.62

*Note:* Table shows HCC untransformed values in pg/mg.

## 4 Discussion

The study objective was to analyze HCCs longitudinally in a large Spanish sample of healthy children, while also examining potential modulatory variables. To this end, pregnant women were recruited, and their sociodemographic and perinatal data were assessed. Their offspring were later evaluated, and HCC was analyzed longitudinally from birth until 7 years of age. The results showed that HCC decreased with age, most steeply early on, with significant HCC variations between children of different ages, from newborns to those aged 4 years. HCC levels were found to stabilize in children aged 4–7 years. Regarding confounder variables, highly stressful events during pregnancy and larger standardized height at birth were associated with lower levels of HCC in newborns. Also, mother BMI and weight, both before and during pregnancy, were negatively associated with HCC at 1 and 2 years of age. Moreover, week of delivery positively correlated with HCC at 3 years of age. Sex differences in HCC were only observed at 3 years of age, with females exhibiting higher levels. Percentile distributions of HCC (pg/mg) were then calculated for newborns, 6‐month‐olds, and 1‐ to 3‐year‐olds.

Comparing the results of the present work with that of the literature, differences and similarities were found. The latter can, however, be explained by the characteristics of previous studies, which must therefore be brought to light. Regarding the relationship between age and child HCC, significant differences were found in this work between consecutive ages, with the variations gradually diminishing with age and ultimately stabilizing between the ages of 4 and 7, when cortisol levels presented minimal variations. These findings are consistent with previous studies that have also reported HCC reduction with age, most steeply early on (Dettenborn et al. 2012; Karlén et al. 2013; Simmons et al. 2019), followed by stabilization between 4 and 7 years (Gerber et al. 2017; Kao et al. 2018; Rippe et al. 2016; Ursache et al. 2017; Vaghri et al. 2013). Furthermore, Noppe et al. (2014) found lower HCC in children aged 4–7 years compared to adults, while no differences were observed between children aged 8–14 years and adults, supporting the classification of children aged 4–7 into the same age group. Contrary to our results, other works have suggested that HCC increases with age (Noppe et al. 2014; Rickmeyer et al. 2017) or decreases in the 4–7 years range (Ling et al. 2019). Those studies, however, used small sample sizes (between 24 and 34 children). Of note, the investigation conducted by Karlén et al. (2013) is particularly relevant regarding the present study as the authors employed a methodology similar to that of this work and their findings align with our results. This is also the type of study advocated by Gray et al. (2018) in their systematic review. Their longitudinal study, which followed a sample of 100 children, demonstrated the following: a decrease in HCC with age between 1 and 8 years (1 > 3 > 5 > 8); a more pronounced decline in the early years; higher and more variable HCC levels in infants (aged 1 year); and increasing HCC stability as children aged, particularly up to 8 years. Consequently, the findings of the present study, along with previous literature, provide evidence that HCC in children decreases with age, at least up to 7 years, and then stabilizes, presenting minimal variations between ages 4 and 7 years.

In relation to the sex of the participants, no differences were found between males and females across all age groups, except for the 3‐year age group, where females exhibited higher HCC levels than males. To the best of our knowledge, no previous studies have analyzed the influence of sex on HCC at 3 years of age or within a similar age range. Previous research involving age ranges similar to our sample (newborns to 5 years) did not find any significant differences in HCC between sexes either (Groeneveld et al. 2013; Karlén et al. 2013; Larsen et al. 2016; Liu et al. 2016; Romero‐González et al. 2018; Vaghri et al. 2013). Other studies have produced different outcomes, showing higher HCC levels in males (Gerber et al. 2017; Grunau et al. 2013; Maurer et al. 2016; Rippe et al. 2016; Villanueva et al. 2017). However, these studies included samples with higher age ranges than ours (primarily children aged 7–12 years). To conclude, most of the evidence suggests an absence of sex differences in the newborn to 5‐year age range, except at 3 years, when females show higher HCC levels.

In the present study, the occurrence of highly stressful events during pregnancy was significantly associated with lower HCC levels only in newborns. No association was observed between 6 months and 3 years, where effect sizes were negligible. In contrast, in children aged 4–7 years, a tendency toward lower HCC levels re‐emerged, with a small effect size that approached statistical significance, suggesting that the association is modest but may be detectable in larger samples. Consistent with our results, lower neonatal HCC has also been reported in relation to other indicators of maternal stress and anxiety during pregnancy, including higher perceived stress, anxiety symptoms, and maternal HCC (Romero‐González et al. 2018; Bryson et al. 2021). With respect to maternal exposure to stressful life events specifically, Broeks et al. (2023) also reported no association between prenatal stressful life events and offspring HCC at 3 months of age, whereas Galbally et al. (2019) found that stressful events at 12 months postpartum were associated with lower offspring HCC at the same time point. Taken together, these findings suggest that maternal stress during pregnancy may influence HCC levels in newborns, although this association appears to disappear thereafter, particularly during the first 3 years of life.

Regarding perinatal variables, birth height SDS was negatively associated with newborn HCC, whereas gestational age at birth was positively correlated with HCC at 3 years of age. To our knowledge, these associations have not been previously examined at these specific ages using SDSs or percentile‐based measures. Contrary to our findings at 1 year, Palmer et al. (2013) reported a negative association between birth height percentile and HCC, whereas Karlén et al. (2013) observed higher HCC among children with non‐appropriate size for gestational age. With respect to gestational age and HCC, previous studies have also reported null associations in newborns and children aged 6 years (Rippe et al. 2016; Romero‐González et al. 2018; Yamada et al. 2007), with the exception of a positive association in newborns reported by Hoffman et al. (2017). Overall, during the first year of life, gestational age at birth appears largely unrelated to HCC, whereas larger birth size adjusted for gestational age is associated with lower HCC. This inverse association may offset the positive link between gestational age and HCC reported by Hoffman et al. (2017), contributing to the predominantly null findings observed across studies.

Furthermore, our study suggested a negative relation between mother prepregnancy and pregnancy BMI and weight and offspring HCC at 1 and 2 years of age, while no relation was found in all the other age groups. To our knowledge, no previous studies have analyzed the influence of maternal weight or BMI on offspring HCC levels, whether before or during pregnancy. However, based on salivary cortisol results, the systematic review of Volqvartz et al. (2023) suggested that maternal BMI does not affect newborn cortisol levels. In their systematic review, Gray et al. (2018) emphasized the need for further research to clarify the role of perinatal variables in both short‐ and long‐term HCC, given the inconsistent findings of previous studies. Our results contribute data on ages that have not been previously studied and suggest that perinatal variables do not influence HCC in children under 1 year and over 3 years of age. Furthermore, when combined with that of previous studies, our results could suggest that the relationship between perinatal variables and HCC in children varies according to age.

Maternal educational attainment, also considered as a potential influential variable, failed to show any association with child HCC across any age group. These results are compatible with that of most studies, as outlined in the systematic reviews by Gray et al. (2018) and R. A. Bates et al. (2022). Most works have failed to identify any significant relationships (Bryson et al. 2019; Gerber et al. 2017; Groeneveld et al. 2013; Karlén et al. 2013; Karlén et al. 2015; Liu et al. 2016; Maurer et al. 2016), while two studies have reported negative correlations (Ursache et al. 2017; Vaghri et al. 2013). Importantly, associations tend to emerge only in studies that deliberately recruit participants across a broad socioeconomic spectrum, including disadvantaged contexts. In non‐disadvantaged populations, compulsory secondary education restricts variability in educational attainment, thereby limiting the ability to detect meaningful differences.

Finally, the composite socioeconomic and psychosocial adversity score was not associated with children's HCC at any age. This null finding aligns with the mixed evidence reported in the systematic review by Bryson et al. (2021), which showed that associations between composite adversity measures and HCC were observed in only about half of the reviewed studies. The absence of associations in the present study, as well as in many others reported in the literature, may reflect the relatively socioeconomically advantaged nature of the samples and the limited variability in adversity exposure, which can hinder the detection of effects that may be more evident in populations experiencing extreme socioeconomic disadvantage (R. A. Bates et al. 2022; Bryson et al. 2021; Simmons et al. 2019; Vaghri et al. 2013).

The results of the present study also provide insight into the levels of HCC observed in children across childhood. When contrasting these values against those of the healthy adult population (Garcia‐León et al. 2018), higher values were found in children compared to adults, especially at early ages. Importantly, elevated HCC may reflect different underlying physiological processes across developmental stages. In adults, higher HCC levels are commonly interpreted in relation to chronic stress exposure and HPA axis dysregulation (R. Bates et al. 2017). In contrast, in young children, elevated HCC may also reflect normative biological demands, given the essential role of glucocorticoids in organ growth, fetal maturation, and development (Waffarn and Davis 2012). Consistent with this view, previous research has encountered a positive relationship between cortisol during pregnancy and offspring health and development, since moderate glucocorticoid levels support an adequate development of cognitive and emotional brain networks (Mariño‐Narváez et al. 2023). Our findings also reflected a reduction in absolute HCC levels as children grow. Two possible reasons can be advanced for this: the fact that organ growth and general development require less cortisol, and the stabilization of HPA axis hyperresponsiveness to stress in early life as suggested in previous studies (Rochette et al. 2023; Russell et al. 2012; Tollenaar et al. 2012).

Moreover, the theory that stress response systems mature over time is supported by the decreasing HCC standard deviations observed in our findings and the longitudinal study by Karlén et al. (2013). Diminishing variability with age suggests a more consistent and stable stress response as children grow older. Our longitudinal design was essential in capturing these dynamics. Similar trends have been observed in previous investigations using salivary cortisol, which reported high variability in younger children (Shirtcliff et al. 2012; Tollenaar et al. 2012). Additionally, Tollenaar et al. (2010) found that cortisol variability peaks at 5 months of age. While these salivary cortisol studies provide valuable insights, they should be interpreted with caution, as salivary cortisol can be influenced by children's eating and sleeping patterns. In conclusion, along with prior research, our findings suggest stress response stabilization during childhood, reflecting the maturation of the stress response system (HPA axis).

It is worth noting certain study limitations. First, the sample was not representative of socioeconomically disadvantaged or adversity‐exposed populations; therefore, the findings are not generalizable to these groups. Second, the number of participants varied across ages, and the sample sizes within the 4‐ to 7‐year age group were smaller; therefore, the nonsignificant results for this age range should be interpreted with caution, and percentiles were not estimated for these ages. Third, children's weight and height during follow‐up time points were not collected, which would strengthen the contextualization of growth patterns in relation to HCC. Finally, children's prenatal and postnatal exposure to traumatic events was not explicitly assessed; however, the occurrence of highly stressful events reported by mothers during pregnancy was considered. While this measure does not capture trauma exposure comprehensively, such exposures also contributed to variability in HCC levels.

Noteworthy, the ELISA method is less specific than LC–MS/MS and has been shown to overestimate cortisol concentrations in immunoassays, resulting in consistently higher HCC levels (Greff et al. 2019). Consequently, the percentile ranges reported here may shift downward if reanalyzed using LC–MS/MS. Moreover, given the strong correspondence between ELISA‐ and LC–MS/MS‐derived HCC values (*r* ^2^ = 0.88–0.97), future studies should prioritize the development of correction factors to harmonize ELISA results with LC–MS/MS equivalents. This approach would improve cross‐study comparability and data integration while preserving the feasibility of large‐scale research, given the high cost and limited accessibility of LC–MS/MS analyses (Greff et al. 2019; Russell et al. 2015). In addition, future research should examine cortisone levels and the cortisol‐to‐cortisone ratio, as these markers provide valuable complementary information beyond isolated HCC and warrant further investigation (R. A. Bates et al. 2022; Stalder and Kirschbaum 2012).

The present study is part of the Gestastress–Childstress cohort, from which multiple publications addressing distinct research questions on prenatal and postnatal biopsychosocial factors have emerged.

## 5 Conclusion

The present work provides important insights into HCC across childhood. By following children longitudinally from birth to 7 years of age, our findings contribute to a better understanding of how cortisol levels change across early developmental stages. Specifically, the results illustrate a general decrease in HCC levels with age, with a steeper decline in the early years and greater stability observed between 4 and 7 years of age.

These findings contribute to the growing literature on HPA axis functioning during childhood and highlight the importance of considering developmental stages when interpreting HCC levels. In addition, the study provides descriptive information on HCC levels observed in children within this cohort, which may help contextualize findings in future research examining stress‐related biological processes in early life.

Overall, HCC represents a valuable noninvasive biomarker for assessing long‐term HPA axis activity. Longitudinal research designs such as the present study are particularly important for improving our understanding of how stress‐related biological processes evolve during childhood and how early‐life experiences may shape these developmental trajectories.

## Author Contributions

**Miguel Ángel Baos‐González**: conceptualization, methodology, formal analysis, investigation, data curation, writing – original draft. **Javier De Echarri‐Lorente**: formal analysis, investigation, data curation, writing – original draft. **María Ángeles García‐León**: methodology, formal analysis, investigation, writing – review and editing. **Raquel González‐Pérez**: investigation, resources, data curation, supervision. **María Isabel Peralta‐Ramírez**: conceptualization, methodology, investigation, resources, writing – review and editing, supervision, project administration, funding acquisition.

## Funding

This work makes part of the project I+D+i Ref. PID2019‐110115GB‐I00, financed by the Ministry of Science, Innovation and Universities and the State Research Agency 10.13039/501100011033. This publication is part of grant PRE2022‐105035, funded by MCIN/AEI/10.13039/501100011033 and by ESF+; CEX2023‐001312‐M, funded by MCIN/AEI/10.13039/501100011033 and UCE‐PP2023‐11 by the University of Granada; and grant PREP2023‐001385, funded by MCIN/AEI/10.13039/501100011033; and the project I+D+i PID2023‐147631NBI00, financed by MCIN/AEI/10.13039/501100011033.

## Conflicts of Interest

The authors declare no conflicts of interest.

## Supporting information




**Table S1. Pairwise comparisons of log‐transformed HCC**. The table presents the mean differences in log‐transformed HCC between age groups, derived from Tukey post‐hoc analyses following ANOVA. Positive values indicate the magnitude of the decline between earlier and later developmental stages. Asterisks denote statistical significance: *p < .05.

## Data Availability

Anonymous data are available upon request to the first author (mbaos@ugr.es).
