# Supplementary material for: Hair Cortisol Concentrations in Children: A Longitudinal Analysis Across Childhood
Source: Dev Psychobiol. 2026 Apr 19;68:e70154. doi: 10.1002/dev.70154 (PMC13092996; doi:10.1002/dev.70154)
Supplement: Supplementary file 1 — Table S1. Pairwise comparisons of log‐transformed HCC. The table presents the mean differences in log‐transformed HCC between age groups, derived from Tukey post‐hoc analyses following ANOVA. Positive values indicate the magnitude of the decline between earlier and later developmental stages. Asterisks denote statistical significance: *p < .05. [file DEV-68-e70154-s001.docx]

**Supplementary Material**

**Table S1.**

*Pairwise comparisons of log-transformed HCC*

|  | **Newborns** | **6 months** | **1 year** | **2 years** | **3 years** | **Over 4 years** |
| --- | --- | --- | --- | --- | --- | --- |
| **Newborns** |  | 1.42* | 1.73* | 1.97* | 2.16* | 2.52* |
| **6 months** |  |  | 0.31* | 0.55* | 0.74* | 1.10* |
| **1 year** |  |  |  | 0.23 | 0.42* | 0.79* |
| **2 years** |  |  |  |  | 0.19 | 0.55* |
| **3 years** |  |  |  |  |  | 0.36* |

*Note:* table shows mean differences based on LN-transformed data derived from Tukey post-hoc test. Asterisks indicate significant values: *p < .05.
